# Synthesis and Characterization of Organo-Soluble Polyimides Based on Polycondensation Chemistry of Fluorene-Containing Dianhydride and Amide-Bridged Diamines with Good Optical Transparency and Glass Transition Temperatures over 400 °C

**DOI:** 10.3390/polym15173549

**Published:** 2023-08-26

**Authors:** Xi Ren, Zhenzhong Wang, Zhibin He, Changxu Yang, Yuexin Qi, Shujun Han, Shujing Chen, Haifeng Yu, Jingang Liu

**Affiliations:** 1 Engineering Research Center of Ministry of Education for Geological Carbon Storage and Low Carbon Utilization of Resources, School of Materials Science and Technology, China University of Geosciences, Beijing 100083, China; renxi@email.cugb.edu.cn (X.R.); wzz0808@163.com (Z.W.); 2103220040@email.cugb.edu.cn (C.Y.); qiyuexin1004@163.com (Y.Q.); 15966200097@163.com (S.H.); chenshujing@cugb.edu.cn (S.C.); 2 School of Material Science and Engineering, Key Laboratory of Polymer Chemistry and Physics of Ministry of Education, Peking University, Beijing 100871, China; zb.he@stu.pku.edu.cn (Z.H.); yuhaifeng@pku.edu.cn (H.Y.)

**Keywords:** polyimide film, fluorene, amide, solution processability, optical transparency, thermal properties

## Abstract

Polymeric optical films with light colors, good optical transparency and high thermal resistance have gained increasing attention in advanced optoelectronic areas in recent years. However, it is somewhat inter-conflicting for achieving the good optical properties to the conventional thermal resistant polymers, such as the standard aromatic polyimide (PI) films, which are well known for the excellent combined properties and also the deep colors. In this work, a series of wholly aromatic PI films were prepared via the polycondensation chemistry of one fluorene-containing dianhydride, 9,9-bis(3,4-dicarboxyphenyl)fluorene dianhydride (FDAn) and several aromatic diamines with amide linkages in the main chain, including 9,9-bis [4-(4-aminobenzamide)phenyl]fluorene (FDAADA), 2,2′-bis(trifluoromethyl)-4,4′-bis[4-(4-aminobenzamide)] biphenyl (ABTFMB), and 2,2′-bis(trifluoromethyl)-4,4′-bis[4-(4-amino-3-methyl)benzamide] biphenyl (MABTFMB). The derived FLPI-1 (FDAn-FDAADA), FLPI-2 (FDAn-ABTFMB) and FLPI-3 (FDAn-MABTFMB) resins showed good solubility in the polar aprotic solvents, such as N-methyl-2-pyrrolidone (NMP), N,N-dimethylacetamide (DMAc) and dimethyl sulfoxide (DMSO). The solution-processing FDAn-PI films exhibited good optical transmittance over 80.0% at a wavelength of 500 nm (T_500_), yellow indices (b*) in the range of 1.01–5.20, and haze values lower than 1.0%. In addition, the FDAn-PI films showed low optical retardance with optical retardation (R_th_) values in the range of 31.7–390.6 nm. At the same time, the FDAn-PI films exhibited extremely high glass transition temperatures (T_g_) over 420 °C according to dynamic mechanical analysis (DMA) tests. The FDAn-PI films showed good dimensional stability at elevated temperatures with linear coefficients of thermal expansion (CTE) in the range of (31.8–45.8) × 10^−6^/K.

## 1. Introduction

It has been more than 100 years since the first demonstration that polymers were covalently linked macromolecules instead of the colloidal systems of aggregates of smaller organic molecules by Hermann Staudinger in 1920 [1]. Meanwhile, it has been 50 years since Hermann Staudinger was awarded the Nobel Prize in Chemistry in 1953 for discoveries in the field of macromolecular chemistry [2]. This theory is also of great significance for the birth of polyimide (PI) materials, the focus of the current paper. Inspired by the macromolecular chemistry theory, DuPont company (Wilmington, DE, USA) launched a basic research program in 1926 to establish and discover new scientific facts [3]. The unremitting efforts of generations of DuPont researchers, including the outstanding contribution of Wallace H Carothers to the foundation and experimental theory of polycondensation chemistry [4], the achievements of his colleague Paul J. Glory in polymer physics [5] and the unremitting exploration of Sroog and coworkers in experiments [6,7], contributed to the birth and commercialization of PI materials in the early 1960s [8]. Table 1 summarizes the representative people, the achievements and the research and development (R&D) related to PI materials.

During the half-century research and development (R&D) activities in PI materials since commercialization in the 1960s, the properties and manufacturing processes of the PIs have been continuously perfected and now PIs have been widely used in modern industry and are well known as the “problem solvers” [11,12,13,14,15]. There have been several golden periods for promoting the rapid development of the PIs. The first stage (1960s~1970s) was featured by the strong driving of the aerospace and weapon industry [16]. The second stage (1970s~1990s) was characterized by the requirements of integrated circuit (IC) industry. The third stage (1990s~now) witnessed the forceful demanding of the advanced PI materials in optoelectronic and information fields. It is different from the previous two stages that promoting the rapid R&D of advanced PIs, the third stage, that is the optoelectronic field, requires PI materials to have excellent thermal stability, high temperature dimensional stability, excellent dielectric and mechanical properties, and at the same times often requires good optical transparency. As we know, the standard wholly aromatic PIs, including the Kapton^®^ film (DuPont, USA) derived from polypyromellitic dianhydride (PMDA) and 4,4′-oxydianiline (ODA), the Upilex^®^-S film (Ube, Japan) from 3,3′,4,4′-biphenylene tetracarboxylic dianhydride (sBPDA) and *para*-phenylenediamine (PDA), and the Upilex^®^-R film (Ube, Japan) based on sBPDA and ODA are usually called “golden films” due to the deep-yellow to brown colors [17]. The colored appearance and poor optical transparency in the ultraviolet–visible light region greatly limited the applications of conventional PI films in the optoelectronic fabrications [18]. Thus, the R&D of colorless PI (CPI) or light-colored and transparent PI films have been becoming one of the most important hot topics in the development of advanced functional PI films [19,20,21,22].

To date, for the sake of the optical transparency of the PI films, various methodologies have been endeavored. All of the modifications are basically based on the well-established charge transfer (CT) theory which ascribed the coloration of standard PI films to the intra- and intermolecular CT interactions occurred from the electron-donating diamine moiety to the electron-receiving dianhydride units [23,24,25,26]. During the transition courses of the excited electrons from the highest occupied molecular orbital (HOMO) in the diamine units to the lowest unoccupied molecular orbital (LUMO) in the dianhydride units, the light absorption edges of the PI films shifted to the longer wavelength higher than 400 nm, that is the visible light region. Thus, the PI films usually exhibited colored appearance [23]. Thus, the CT interactions in the PIs should be eliminated or prohibited in order to endow the film colorless or pale-color features. Generally, four procedures are often used to prohibit the CT effects in PI films, including the introduction of non-conjugated molecular skeletons (aliphatic or alicyclic units, etc.) [27], introduction of highly electronegative groups (fluoro-containing groups, etc.) [28], introduction of substituents with bulky molecular volumes (fluorene, sulfone groups, etc.) [29], and introduction of asymmetrical groups in the main chain or side chain of the PIs. Choice of the appropriate modification procedures mentioned above is highly dependent on the specific property requirements of the practical optoelectronic applications. Considering the facts that high-temperature fabrication up to 260 °C or higher are often required in the manufacturing or reliability tests of the optoelectronic devices [30], the CPI films should possess high thermal resistance. High T_g_ and low CTE are often simultaneously require for the CPI films. This is often accompanied the inter-conflicting molecular design for the high-temperature resistant CPI films because the procedures enhancing the thermal stability of the CPI films often deteriorate the optical transparency of the afforded polymers. Li and coworkers reported the heat-resistant CPI films based on the benzimidazole diamines and fluoro-containing dianhydride, 2,2′-bis(3,4-dicarboxyphenyl) hexafluoropropane dianhydride (6FDA) and 1,2,4,5-cyclohexanetetracarboxylic dianhydride (HPMDA) [31]. The afforded CPI films had high T_g_ values in the range of 345–402 °C and the CTE values of (42.0–49.0) × 10^−6^/K. However, the 6FDA-based CPI films showed optical transmittance at a wavelength of 400 nm (T_400_) of 10~38%, while the HPMDA-based ones had values of 80–81%. Yan et al. reported the CPI films with high T_g_ from asymmetric twisted benzimidazole diamines and 6FDA or HPMDA [32]. The HPMDA-derived CPI films had T_g_ up to 409–421 °C and the CTE values of (48.0–50.2) × 10^−6^/K with T_400_ values in the range of 76–80%. Hasegawa and coworkers reported the strategy to develop HPMDA-based semi-alicyclic CPI films with low-CTE features via incorporation of the rigid-rod amide-containing units into the PIs [33]. Inspired by this strategy, a series of amide-containing CPI films with both of high optical transparency, high-T_g_ and low-CTE characteristics were developed in the literature [34,35]. In particular, very recently, Li and coworkers reported the semi-alicyclic CPI films based on the alicyclic dianhydrides and the benzimidazole diamine containing biamide units [36]. The derived CPI films had T_g_ up to 404 °C and the CTE as low as 22.0 × 10^−6^/K.

Although the semi-alicyclic CPI films with good comprehensive properties have been developed in the literature, the wholly aromatic PI films still showed great promise as the components for advanced optoelectronic applications due to the good solvent resistance, high tensile strength and modulus. In the current work, such PI films were endeavored to be developed based on the fluorene-containing dianhydride, 9,9-bis(3,4-dicarboxyphenyl)fluorene dianhydride (FDAn) and the amide-containing diamines. Fluorene groups represent a class of rigid and fused-ring structure with high thermal resistance and bulky molecular volumes [37]. As for the optical and dielectric features, the fluorene groups can usually endow the derived polymer films with good optical transparency, high refractive indices, low birefringence, low optical retardation, and low dielectric constants [38,39,40]. Fluorene substituents were usually incorporated into the molecular structure of aromatic diamines and few works have been reported on the CPI films derived from fluorene-containing dianhydrides in the literature to the best of our knowledge. The effects of the fluorene and amide units on the thermal and optical properties of the derived PI films were investigated in detail in the current work.

## 2. Materials and Methods

### 2.1. Materials

9,9-Bis(3,4-dicarboxyphenyl)fluorene dianhydride (FDAn) and 2,2′-bis(3,4-dicarboxyphenyl)hexafluoropropane dianhydride (6FDA) were purchased from ChinaTech Chem. Co. Ltd., Tianjin, China and recrystallized from acetic anhydride with the de-coloring agents of active carbon power and then dried at 180 °C in vacuo for 10 h prior to use. The aromatic diamines, including 9,9-bis[4-(4-aminobenzamide)phenyl] fluorene (FDAADA), 2,2′-bis(trifluoromethyl)-4,4′-bis[4-(4-aminobenzamide)]biphenyl (ABTFMB) and 2,2′-bis(trifluoromethyl)-4,4′-bis[4-(4-amino-3-methyl)benzamide] biphenyl (MABTFMB) were synthesized and purified by recrystallization from absolute ethanol with the de-coloring agents of active carbon power in our laboratory. The purities of the aromatic diamines were all higher than 99.0% for the polycondensation. The ultra-dry DMAc and N-methyl-2-pyrrolidone (NMP) solvents with a water content lower than 50 ppm were purchased from InnoChem Science & Technology Co., Ltd., Beijing, China and used directly. Toluene and the other commercially available reagents with the analytical purity were used as received.

### 2.2. Characterization Methods

The inherent viscosities ([η]_inh_) of the soluble PI resins were detected with an Ubbelohde viscometer (Mitong Electromechanical Tech. Co., Ltd., Shanghai, China) with a NMP solution (solid content: 0.5 g/dL) at room temperature of 25 °C. Number average molecular mass (M_n_) and weight average molecular mass (M_w_) of the soluble PI resins were obtained by the gel permeation chromatography (GPC) measurement with the apparatus developed in Shimadzu (Kyoto, Japan). The HPLC grade of NMP was used as the mobile phase. The polydispersity index (PDI) of the molecular weights were calculated as: PDI = M_w_/M_n_. Hydrogen nuclear magnetic resonance (^1^H-NMR) of the soluble PI resins was conducted with the AV 400 spectrometer developed by Bruker Optics (Ettlingen, Germany) at the frequency of 400 MHz. The ^1^H-NMR solvent was deuterated dimethyl sulfoxide (DMSO-d_6_). Solubility of the PI resins was determined as follows: Into a 50 mL glass bottle was added 1.0 g of the PI resin and 9.0 g of the tested solvent at room temperature to afford a mixture with a solid content of 10 wt%. The mixture was mechanically stirred for 24 h. The solubility was visually determined as three grades: completely soluble (++), partially soluble (+), and insoluble (−).

Fourier transform infrared (FTIR) spectra of the FDAn-PI films were detected on a Shimadzu Iraffinity-1S FTIR spectrometer (Kyoto, Japan) with a wavenumber of 4000–400 cm^−1^. Ultraviolet–visible (UV–Vis) spectra of the FDAn-PI films were measured with a U-3210 spectrophotometer developed by Hitachi (Tokyo, Japan). Wide-angle X-ray diffractions (XRD) of the FDAn-PI films were conducted on a D/max-2500 X-ray diffractometer developed by Rigaku (Tokyo, Japan). The optical parameters of the PI films were detected on a color i7 spectrophotometer developed by X-rite (Grand Rapids, MI, USA) using a CIE Lab equation. L* stands for the lightness from 0 (black) to 100 (white). A positive a* stands for a red color, and a negative one is a green color. A positive b* stands for a yellow color, and a negative one is a blue color. The in-plane refractive indices (n_TE_) and out-of-plane refractive indices (n_TM_) of the PI films were measured with a Metricon Model 2010/M prism coupler at a wavelength of 632.8 nm. The average refractive indices (n_av_) were expressed as n_av_ = [(2n_TE_^2^ + n_TM_^2^)/3]^1/2^. The birefringence (Δn) of the CPI films were calculated as Δn = n_TE-_n_TM_ and the R_th_ values were calculated as R_th_ = Δn × d, where d stands for the thickness of the CPI films. Thermogravimetric analyses (TGA) of the PI films were conducted on a Q50 thermal analysis system developed by TA Instruments (New Castle, DL, USA) at a heating rate of 20 °C/min in nitrogen. Dynamic mechanical analysis (DMA) was recorded on a Q800 thermal analysis system by TA Instruments (New Castle, DL, USA) at a heating rate of 5 °C/min and a frequency of 1Hz in nitrogen. Thermo-mechanical analyses (TMA) of the PI films were tested on a TMA402F3 thermal analysis system developed by NETZSCH (Selb, Germany) with a heating rate of 5 °C/min in nitrogen. The CTE values were detected from 50 °C to 250 °C. The tensile strength (T_s_), elongations at break (E_b_), and tensile modulus (T_m_) of the PI films were detected with a 3365 Tensile Apparatus developed by Instron (Norwood, MA, USA) with 80 mm × 10 mm × 0.05 mm specimens.

### 2.3. PI resin Synthesis and the Film Preparation

The FDAn-PI resins were synthesized from FDAn and the diamines by the chemical imidization pathway. FLPI-1 (FDAn-FDAADA) was used to illustrate the detailed preparation procedure. The relative humidity of the environments for the polycondensation reaction was controlled to be lower than 50%. Then, FDAADA (29.3340 g, 0.05 mol) and ultra-dry DMAc (150.0 g) were added into a 500 mL glass vessel with a mechanical stirrer, a nitrogen inlet, and a cold water bath was added. The clear diamine solution was obtained after stirring at room temperature for 10 min under nitrogen. FDAn dianhydride (22.9210 g, 0.05 mol) was rapidly added and an additional DMAc (59.0 g) was added together. A solid content of 20 wt% was achieved for the polymerization mixture. After 3h, the cold bath was removed and the temperature rose to room temperature (25 °C) after the viscous solution was stirred for 3 h. Then, the reaction was continued until the total polymerization time reached 24 h. To the pale-yellow and viscous poly(amic acid) (PAA) solution was added the dehydrating agent of acetic anhydride (51.0 g, 0.5 mol) and the catalyst of pyridine (31.6 g, 0.4 mol) with vigorous stirring. The chemical imidization procedure was performed at room temperature for another 24 h. The reaction mixture was then poured into an excess of aqueous ethanol solution (75 vol%). FLPI-1 resin precipitated from the ethanol solution and was obtained as a silky resin with a pale-yellow appearance. The resin was thoroughly immersed into the ethanol solution and then filtered from the solution. The collected resin was first dried in air environment and then in vacuo at 120 °C for 24 h. The pale-yellow fibrous FLPI-1 resin was finally obtained. Yield: 49.3 g (97.7%). M_n_: 3.17 × 10^4^ g/mol; M_w_: 5.89 × 10^4^ g/mol; PDI: 1.85. ^1^H-NMR (DMSO-d_6_, ppm): 10.34 (s, 2H), 8.07–7.93 (m, 8H), 7.71–7.32 (m, 24H), and 7.13–7.11 (d, 2H).

The other PI resins, including FLPI-2 (FDAn-ABTFMB) and FLPI-3 (FDAn-MABTFMB) were prepared by the similar procedure except FDAADA was changed to ABTFMB for FLPI-2 and MABTFMB for FLPI-3, respectively.

The fully dried FLPI-1 resin was mixed with DMAc at a solid content of 15 wt% at room temperature. The afforded clear and transparent PI solution was filtrated with a 1.0 μm Teflon syringe filter. The PI varnish was de-foamed in vacuo and then cast onto a clean glass with a doctor knife. The wet film was then thermally baking in a clean oven according to the procedure of 80 °C/2 h, 150 °C/1 h, 180 °C/1 h, 200 °C/1 h, and 250 °C/1 h. Then, the obtained glass carrier was cooled to room temperature and immerged into the deionized water. FLPI-1 film peeled off the substrate. The free-standing film was dried in vacuo at 120 °C for 24h. FT-IR (cm^−1^): 1778, 1714, 1668, 1601, 1506, 1360, 1319, 1082, and 731.

The other PI films, including FLPI-2 (FDAn-ABTFMB) and FLPI-3 (FDAn-MABTFMB) were fabricated by the similar procedure.

## 3. Results and Discussion

### 3.1. PI resin Synthesis and Film Preparation

**Figure 1:**
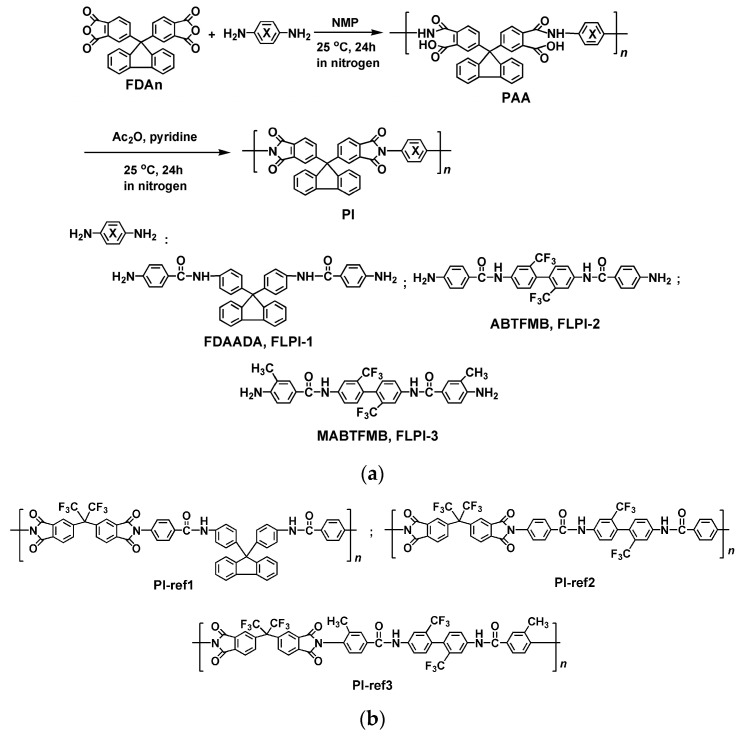
Preparation pathway of the PI resins. (**a**) FDAn-PI (FLPI-1~FLPI-3); (**b**) 6FDA-PI (PI-ref1~PI-ref3).

As presented in Figure 1, three PI resins, FLPI-1 (FDAn-FDAADA), FLPI-2 (FDAn-ABTFMB) and FLPI-3 (FDAn-MABTFMB) were prepared via the two-stage chemical imidization procedure from FDAn dianhydride and different amide-containing aromatic diamines, respectively. The poly(amic acid) (PAA) precursor solutions were in situ dehydrated under the action of acetic anhydride (Ac_2_O) with the catalyst of pyridine to afford the soluble PI solutions. No macroscopical precipitation or gelling phenomena were observed during the imidization process although the existence of the rigid-rod amide units in the molecular structures of the PIs. By similar procedure, three referenced PI resins, including PI-ref1 (6FDA-FDAADA), PI-ref2 (6FDA-ABTFMB) and PI-ref3 (6FDA-MABTFMB) were also prepared from 6FDA and the same diamines.

**Figure 2:**
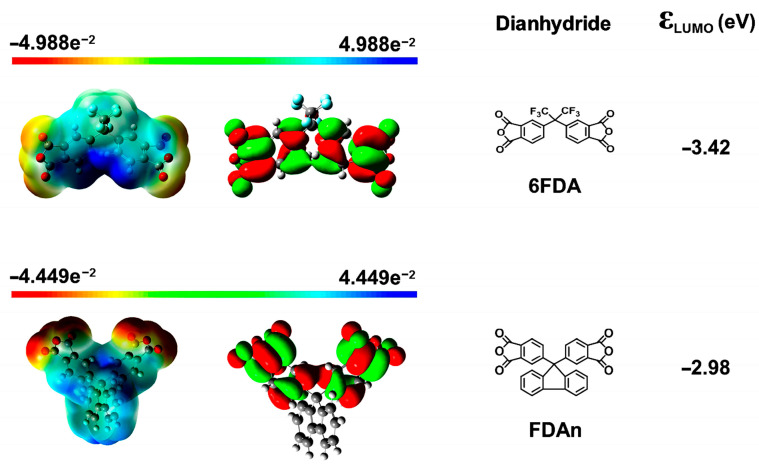
Simulated steric structures and the calculated molecular orbit energies (ε_LUMO_) of the aromatic dianhydrides.

The physical characteristics of the PI resins, including the inherent viscosities ([η]_inh_), molecular weights and the solubility in representative organic solvents are summarized in Table 2. It could be seen from the data that, if the diamine was fixed, the PI resins derived from 6FDA usually exhibited the higher [η]_inh_ and molecular masses. For example, for the same diamine of FDAADA, PI-ref1 (6FDA-FDAADA) had [η]_inh_ and number average molecular mass (M_n_) values of 0.77 dL/g and 3.78 × 10^4^ g/mol, which were higher than those of FLPI-1 derived from FDAn ([η]_inh_ = 0.73 dL/g; M_n_ = 3.17 × 10^4^ g/mol). The same trend was founded for the other two resin systems. This partially indicated the potentially higher polymerization reactivity for the 6FDA compared with FDAn when the spread of the polymer chains (radius of gyration) in the solvent was not concerned in the GPC measurements. In the literature, it has ever been reported that the reactivity of the dianhydride monomers could be roughly estimated via the lowest unoccupied molecular orbital (LUMO) energy levels (ε_LUMO_) simulated by frontier orbital calculations according to the density functional theory (DFT) [41]. For the dianhydride monomer, a lower ε_LUMO_ value usually indicated a higher reactivity of the monomer. The molecular orbit simulation results based on DFT and B3LYP methods with Gaussian 09 software using the 6–311 G(d, p) basis set were shown in Figure 2. 6FDA exhibited a lower ε_LUMO_ value of −3.42 eV, which was a bit lower than that of the FDAn dianhydride (ε_LUMO_ = −2.98 eV). This revealed a higher polymerization reactivity of the 6FDA, which might be due to the existence of highly electron-withdrawing hexafluoroisopropylene units in the compound. Comparatively, the FDAn dianhydride contained the electron-donating fluorene substituents, which increased the electron density on the anhydride groups and further weakened the reactivity with the diamine monomers. Basically, both of the FDAn-PI and 6FDA-PI resins possessed the moderate to high molecular weights, which were quite beneficial for endowing the derived PI films good comprehensive properties.

**Figure 3:**
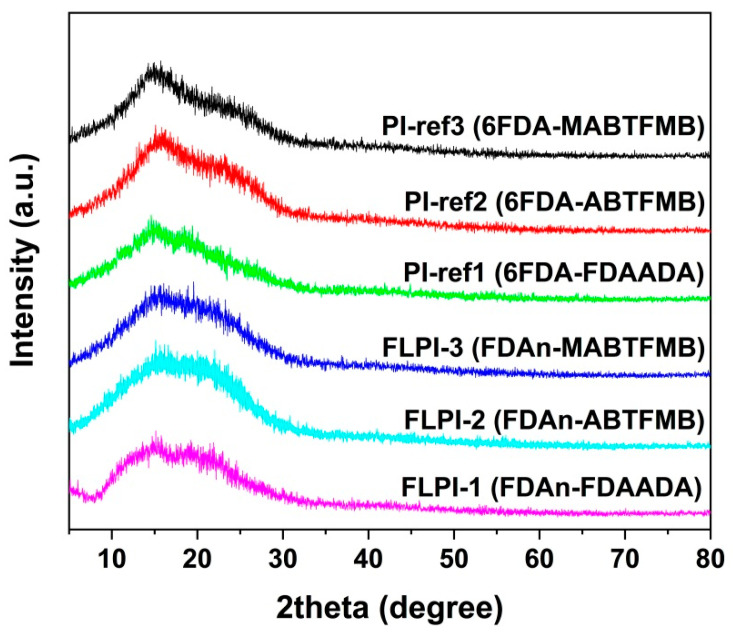
XRD patterns of FDAn-PIs and referenced PIs.

In addition, the developed FDAn-PI resins exhibited excellent solubility in the tested solvents, as shown in Table 2. They were soluble in NMP, DMAc, γ-butyrolactone and other polar aprotic solvents at the solid content of 10 wt% at room temperature. They were also totally soluble in the less-polar cyclopentanone (CPA) except FLPI-2. All the PI resins were partially soluble in chloroform. The good solubility of the PI resins were mainly due to the bulky fluorene or hexafluoroisopropylene units in the dianhydride moiety and the bulky fluorene, trifluoromethyl, or methyl substituents in the diamine moiety. The existence of these bulky groups efficiently decreased the ordered packing of the molecular chains in the PIs, making the polymers exhibit amorphous nature in the XRD measurements (Figure 3). No apparent crystalline peaks were detected for the PI resins in the scattering angle from 5° to 80°. Alternatively, only the wide and blunt peaks were observed in the range of 10–30°.

**Figure 4:**
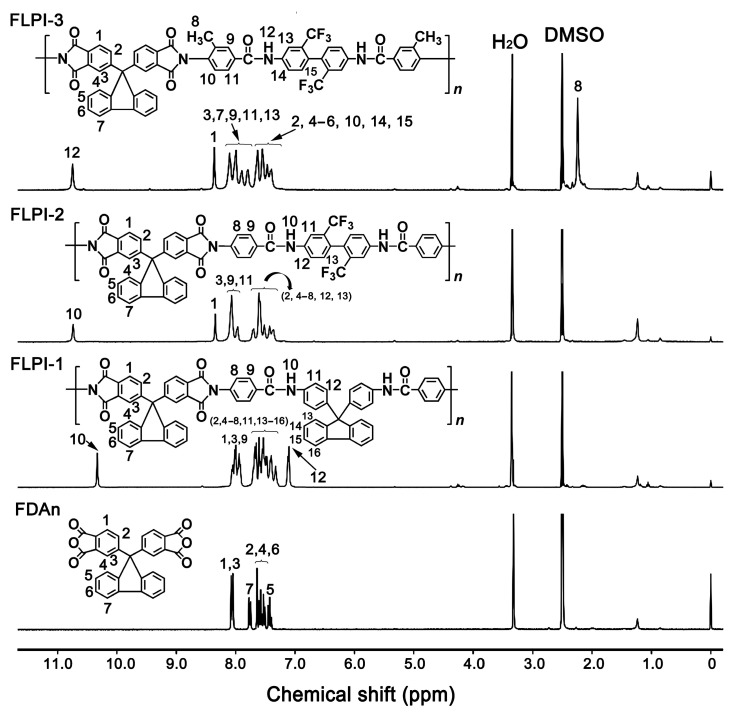
^1^H-NMR spectra of FDAn and the derived FDAn-PI resins.

The FDAn-PI resins were dissolved into deuterated dimethyl sulfoxide (DMSO-d_6_) and the ^1^H-NMR measurements were performed and the results are shown in Figure 4. For all of the FDAn-PIs, the characteristic absorptions of the hydrogen protons in the –CONH– units were observed at the farthest downfield in the spectra. The protons adjacent to the electron-withdrawing imide carbonyl groups (H_1_) revealed the absorptions at the second farthest downfield in the spectra. The aromatic hydrogen protons in the benzene and fluorene rings had resonance in the chemical shift range of 7.0–8.5 ppm, which were similar with those in the FDAn dianhydride. For FLPI-3, the methyl protons exhibited the resonance at the farthest upfield in the spectra. These structural features were in good agreement with the actual structures of the polymers, indicating the successful preparation of the targeted polymers.

A series of PI films were prepared by casting the preimidized PI solutions in DMAc onto glass substrates and then dried from 80 °C to 250 °C to remove the solvents. The low-temperature curing feature of the solution processable PIs is usually very beneficial to maintain the optical transparency of the derived films because it avoids the possible thermal oxidation and discoloration problems caused by high-temperature treatment. In the current work, the light-colored and optically transparent PI films were obtained. The tensile properties, including the tensile strength (T_S_), elongations at break (E_b_) and tensile modulus (T_M_) are shown in Table 3. The PI film had T_S_ values higher than 110 MPa and the T_M_ values in the range of 3.9–5.9 GPa, indicating the flexible and tough nature of the PI films. This is mainly due to the high molecular weights and the rigid-rod amide units in the diamine moieties. Although the PI films showed somewhat low E_b_ values, they could be remedied by the biaxially stretching treatments in the future manufacturing procedure, which has been proven to be highly efficient for improving the E_b_ values of the PI films [42].

**Figure 5:**
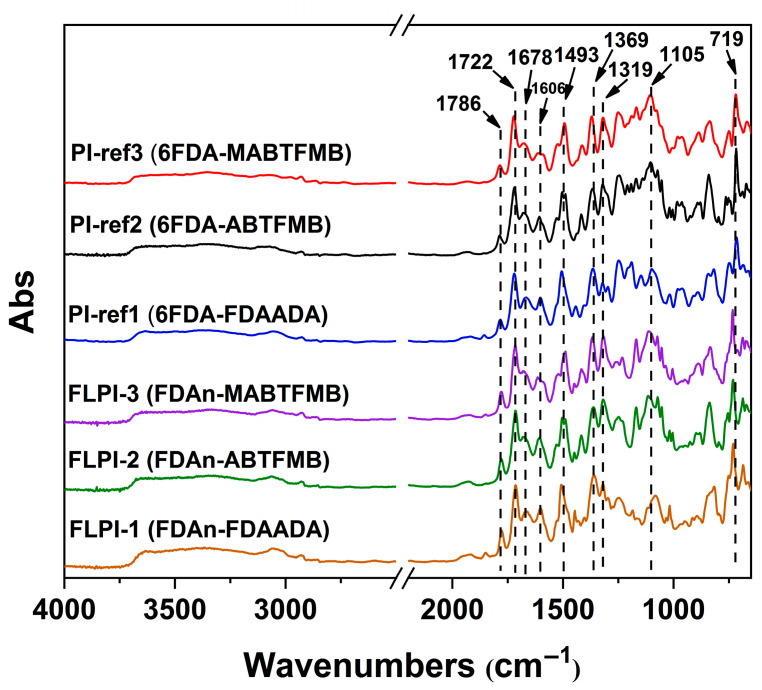
FTIR spectra of FDAn-PIs and referenced PI films.

The chemical structures of the PI films were identified by the FTIR measurements and the results are shown in Figure 5. First, the characteristic absorptions of the imide rings were observed in all the PI films. The imide carbonyl vibrations, including the asymmetrical stretching vibrations around 1786 cm^−1^, the symmetrical ones around 1722 cm^−1^, and the bending vibrations around 719 cm^−1^ were all observed. The imide C–N stretching around 1369 cm^−1^ was also detected. In addition, the amide (–CONH–) carbonyl stretching vibrations around 1678 cm^−1^ and the C=C stretching vibrations in phenyl around 1493 cm^−1^ and were also found. There are two kinds of –CF_3_ groups in the 6FDA-PIs, including the ones in the aliphatic hexafluoroisopropylene linkages in the dianhydride moiety and the ones attached to the benzene rings in the diamine moiety (PI-ref2 and PI-ref3). The former and the latter C-F bonds had characteristic stretching vibrations around 1105 cm^−1^ and 1319 cm^−1^, respectively. However, for the FDAn-PIs, only the absorptions of the latter C-F bonds were detected. These structural features were also in consistent with the actual structures of the polymers.

### 3.2. Thermal Properties

**Figure 6:**
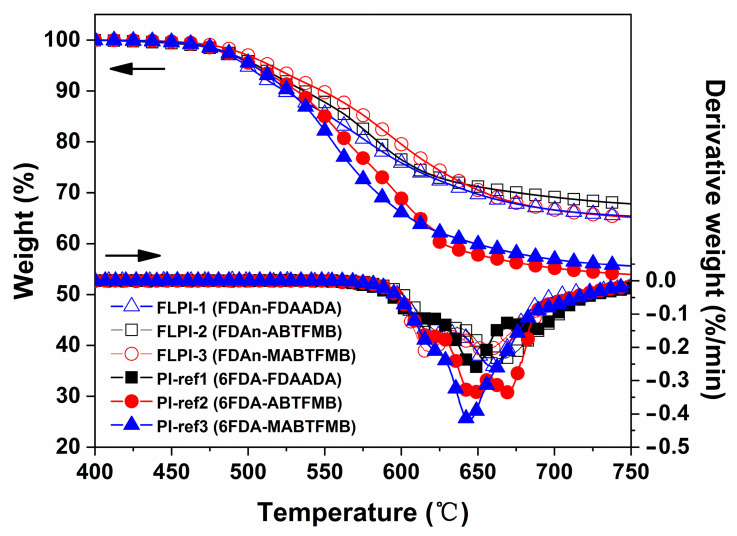
TGA and DTG curves of FDAn-PIs and referenced PI films.

The thermal data of the FDAn-PI films were detected by TGA, DMA and TMA measurements and the results are tabulated in Table 3. Figure 6 presents the TGA and DTG plots of the PI films. The samples exhibited good thermal stability up to 500 °C, after which the PI films start decomposing. The PI films had 5% weight loss temperature (T_5%_) in the range of 498.7–514.4 °C, which were comparable to those of the referenced 6FDA-PI samples. For example, FLPI-1 had the T_5%_ value of 505.5 °C, which was nearly 5 °C higher than that of the analogous PI-ref1 sample (T_5%_ = 500.7 °C). The FDAn-PIs showed the most rapid decomposition at approximately 575.0–591.7 °C according to the DTG plots of the samples, which were obviously higher than those of the 6FDA-PI counterparts. For FLPI-1, the maximum decomposition rate (Tmax) occurred at 582.6 °C, which was 23.0 °C higher than that for PI-1. This indicates that the fluorene units exhibited higher thermal endurance at elevated temperatures, which might be due to the high aromatic contents in the fluorene substituents. Due to the superior thermal resistance of the fluorene units, the FDAn-PI samples had a residual weight ratio higher than 65.0% at 750 °C (R_w750_), which was also higher than those of the corresponding 6FDA-PI samples.

**Figure 7:**
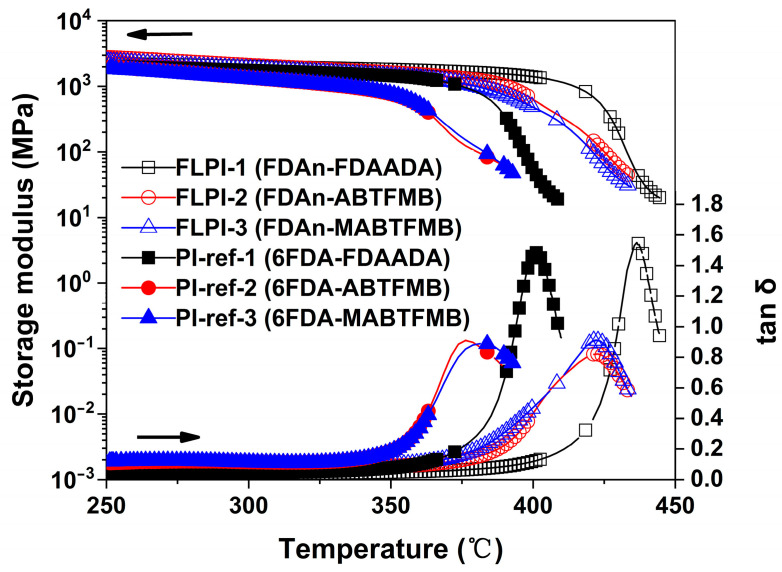
DMA curves of FDAn-PIs and referenced PI films.

Figure 7 depicts the DMA plots of the PI films. For the FDAn-PI films, all the samples maintained the initial storage modulus up to 350 °C, after which the modulus gradually decreased. With the increasing test temperatures, the PI samples revealed the peak temperatures in the tanδ plots, which were assigned as the T_g_ values of the polymers. As shown in the figure and Table 4, the FDAn-PI had T_g_ values in the range of 422.2–436.4 °C, which were more than 35 °C higher than those of the 6FDA-PIs. For example, FLPI-1 exhibited the T_g_ value of 436.4 °C, which was 35.1 °C higher than that of the analogous PI-ref1 (T_g_ = 401.3 °C). FLPI-2 and FLPI-3 had T_g_ values of 422.6 °C and 422.2 °C, which were 46.3 °C and 40.8 °C higher than those of the analogous PI-ref2 (T_g_ = 376.3 °C) and PI-ref3 (T_g_ = 381.4 °C), respectively. Undoubtedly, the bulky molecular structure of the fluorene units efficiently prohibited the free motion of the molecular chain segments of the PIs; thus obviously improved the Tg values of the PIs. Comparatively, the flexible hexafluoroisopropylene units in 6FDA were prone to move at elevated temperatures. In the same PI system, the sample derived from fluorene-containing FDAADA diamine had the highest T_g_ value due to the existence of the bulky fluorene units in the diamine. FLPI-1 and PI-1 all had the highest T_g_ values in the individual system.

**Figure 8:**
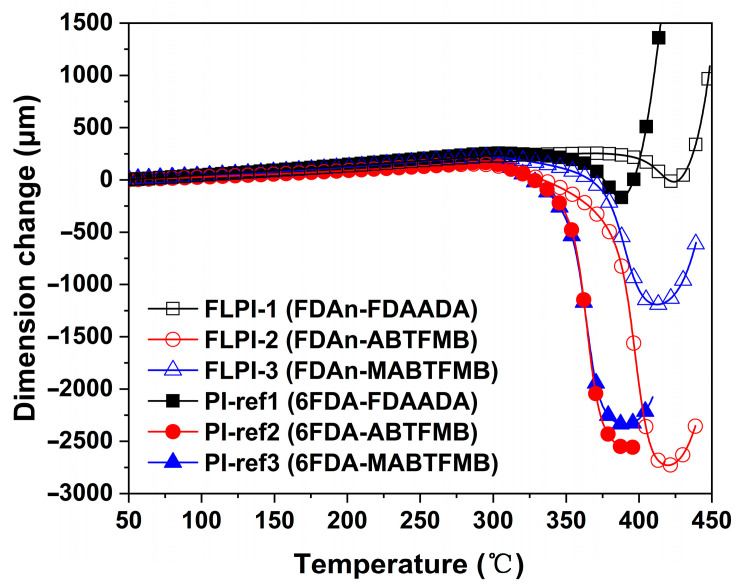
TMA curves of FDAn-PIs and referenced PI films.

Figure 8 presents the TMA plots of the PI samples. Before the glass transition temperatures of the PIs, all the samples showed linear thermal expansion behaviors. From 50 °C to 250 °C, the FDAn-PI films had CTE values of 45.8 × 10^−6^/K for FLPI-1, 31.8 × 10^−6^/K for FLPI-2, and 42.8 × 10^−6^/K for FLPI-3. Basically, the FDAn-PI films had moderate CTE values. FLPI-2 exhibited the lowest CTE value in the system, which might be due the rigid amide and biphenyl units in the ABTFMB moiety. For the structurally analogous FLPI-3, the CTE value increased to 42.8 × 10^−6^/K due to the flexible methyl substituents in the MABTFMB units. For the 6FDA-PI systems, the PI-ref1 and PI-ref2 films exhibited higher CTE values than those of the FLPI-1 and FLPI-2, while PI-ref3 had the lower one than that of FLPI-3. For all of the samples, when the test temperatures reached the T_g_ values of the polymers, the films showed obviously shrinking behaviors, indicating the rearrangements of the molecular chains in the PIs. When the molecular chains alignments finished, the PI films expanded again. The transition temperature peaks could also be assigned as the T_g_ values of the PI samples, which were similar with those recorded by the DMA measurements.

In summary, the currently developed fluorene-containing PI films exhibited good thermal resistance with T_g_ over 420 °C and moderate CTE values and might endure the possible severe high-temperature environments in practical applications.

### 3.3. Optical Properties

**Figure 9:**
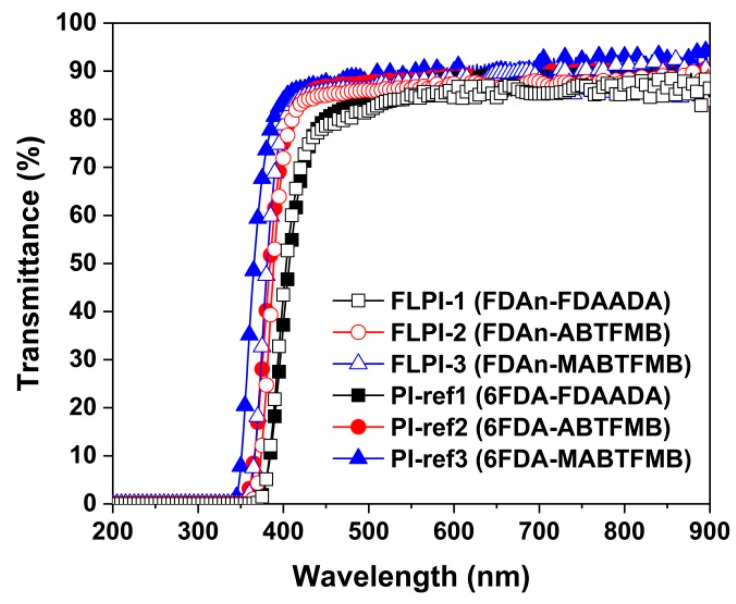
UV–Vis spectra of FDAn-PIs and referenced PI films.

The optical data of the PI films were evaluated by ultraviolet–visible (UV–Vis) spectra, refractive indices and CIE Lab color parameters measurements and the data are tabulated in Table 4. Figure 9 depicts the UV–Vis spectra of the PI films. Comparatively, the FDAn-PI films showed inferior optical transparency to those of the corresponding 6FDA-PI samples. For example, the FDAn-PI film had a UV cutoff wavelength (λ_cut_) from 348 nm to 364 nm, while the 6FDA-PIs had a λ_cut_ of 336–365 nm, indicating the wider optically transparent wavelength range for the latter polymers. As for the optical transmittance values of the PI films, FLPI-1 exhibited optical transmittance at wavelengths of 450 nm (T_450_) and 500 nm (T_500_), with values of 78.6% and 81.8%, respectively, which were slightly lower than those of the PI-ref1 (T_450_ = 80.0%; T_500_ = 84.5%). Among the individual PI systems, the PI films derived from MABTFMB had the highest T_450_ and T_500_ values. For example, FLPI-3 and PI-ref3 exhibited the highest T450 values of 85.7% and 87.6%, respectively, which might be due to the synergic effects of methyl and trifluoromethyl substituents. The non-fluorinated FLPI-1 film had the worst optical transmittance in the systems.

**Figure 10:**
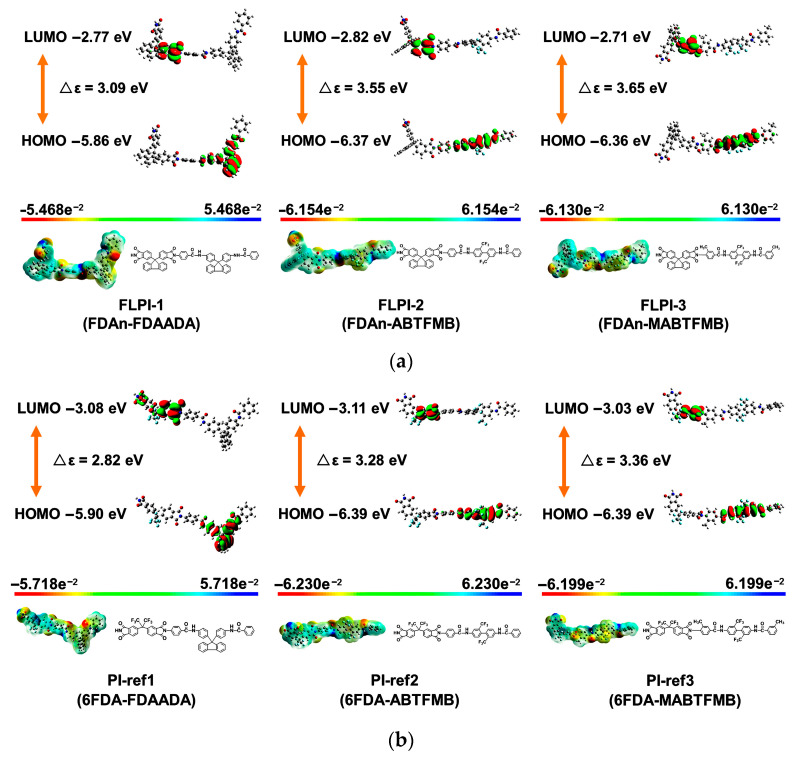
Simulated steric structures and the calculated molecular orbit energy gaps (Δε) of the PIs. (**a**) FLPI samples; (**b**) PI-ref samples.

The experimental optical property results could be further verified by the frontier orbit simulation based on the DFT theory, as shown in Figure 10. It has been well established that the optical transparency of the donor-acceptor (D-A) polymers, such as PIs could be estimated by the energy gaps (Δε) between the HOMO and LUMO energy levels in the polymers [43]. The larger the absolute Δε values, the less the degree of charge transfer (CT) interactions within the PI molecular chains, and the better the optical transmittance of the derived PI films. As shown in Figure 10, the LUMO, HOMO energy levels and the calculated Δε values (|HOMO-LUMO|) were marked. The PI film showed increasing Δε values with a trend of PI-ref1 (2.82 eV) < FLPI-1 (3.09 eV) < PI-ref2 (3.28 eV) < PI-ref3 (3.36 eV) < FLPI-2 (3.55 eV) ≈ FLPI-3 (3.65 eV). This trend means that the optical transmittances of the PI films would show the same order. In practice, PI-ref1 and FLPI-1 PI films indeed exhibited the relatively worse optical transparency in the systems (Table 4).

The refractive indices, including the n_TE_ and n_TM_ of the PI films were further tested and the n_av_, Δn and R_th_ values were calculated. The results are summarized in Table 4. As we know, the refractive indices of the polymeric optical films are mainly influenced by the molar refraction (P) and molar volume (V) of the structural units according to the Lorentz-Lorenz equations [44]. Higher P/V values usually afford the polymers with higher refractive indices, and vice versa. Fluorene groups usually exhibit high P/V value due to extremely high P values although the high V values at the same time. Contrarily, the hexafluoroisopropylene and trifluoromethyl groups usually showed low P/V values; thus; are usually used to develop PI films with low refractive indices. The data shown in Table 4 indicate that the FLAn-PI films exhibited higher n_av_ values than those of the 6FDA-PIs. FLPI-1 exhibited the highest n_av_ value of 1.6842 at a wavelength of 632.8 nm, which was obviously higher than that of the analogous PI-ref1 film (n_av_ = 1.6357). In addition, the PIs derived from the fluorene-containing FDAADA diamine had the highest n_av_ values in the systems due to the high contents of fluorene components. On the contrary, the PIs based on MABTFMB had the lowest n_av_ values in the systems due to the increased molar volumes caused by the pendant methyl substituents. With respect to the birefringence (Δn) of the PI films, FLPI-1 with the highest fluorene components had the lowest Δn value of 0.00317. PI-ref1 derived from FDAADA exhibited the second lowest Δn value of 0.01131. While the PIs derived from ABTFMB had the highest Δn value of 0.05970 for PI-ref2 and 0.03906 for FLPI-2, respectively. The higher Δn values of the ABTFMB-based PIs were due to the relatively higher intermolecular chain packing density of the PIs caused by the polar –CONH– units [45]. The lowest Δn value of FLPI-1 resulted in the lowest optical retardation (R_th_) for the polymer, which was quite important for the practical applications [46]. The R_th_ value of 31.7 nm for the fluorene-containing FLPI-1 was comparable or lower than those of the low-R_th_ CPI films reported in the literature [47,48]. It has been well established in the literature that the R_th_ values of the polymer films are usually strongly influenced by the orientation state and aggregation structure of the polymer chains in the films [49,50]. Generally, polymer films with lower CTE values often exhibit higher Δn and R_th_ values. In the current work, for the fluorene-containing PI films, the CTE values decreased in the order of FLPI-1 > FLPI-3 > FLPI-2 and the R_th_ values decreased in the order of FLPI-1 < FLPI-3 < FLPI-2. This shows good consistency with the literature. However, for the relationship of the R_th_ and T_g_ values of the PI films, it looks more complicated. This is mainly due to the multiple factors affecting the T_g_ of the polymers, mostly the electron effects and steric effects for the molecular chain interactions in the polymers. In the current work, FLPI-1 with high contents of bulky fluorene substituents simultaneously had the highest T_g_ and the lowest R_th_ values. Meanwhile, the FLPI-3 showed obviously lower R_th_ value than that of FLPI-2 although they had very similar T_g_ values. For the PI films without fluorene units, such as PI-ref2 and PI-ref3, the former polymer exhibited a lower T_g_, but a higher R_th_ value than those of the latter polymer. This indicates that it is difficult to find a clear interaction relationship between the T_g_ and R_th_ in the PI films.

**Figure 11:**
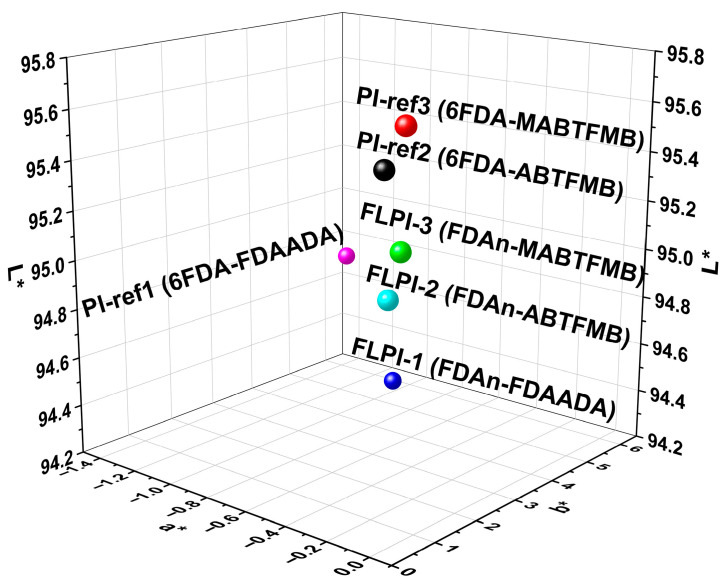
CIE Lab color parameters of FDAn-PIs and referenced PI films.

At last, the optical properties of the PI films were evaluated by the CIE color parameters, as shown in Table 4 and Figure 11. Basically, the PI films derived from the fluorinated diamines all had lower yellow indices (b*) than those from the unfluorinated ones (FLPI-1 and PI-ref1). The ABTFMB- and MABTFMB-derived PI films had low b* values of approximately 1.0, while the FDAADA-derived ones exhibited the b* value around 5.0. This is due to the inhibition of CT interactions by the highly electronegative –CF_3_ groups in the fluorinated diamines. All the PI films had haze values below 1.0%, which was quite beneficial for the applications in optoelectronic fields.

## 4. Conclusions

Wholly aromatic PI films with high optical transparency, low optical retardation, and T_g_ values over 400 °C were developed in the current work. The specific physical and chemical features of the fluorene groups endowed the derived PI films good comprehensive properties. For example, the FLPI-1 film with the highest fluorene contents exhibited good combined properties, including a T_g_ value of 436.4 °C, a CTE value of 45.8 × 10^−6^/K, a T_500_ value of 81.8%, a b* value of 5.20, a haze value of 0.73, a n_AV_ value of 1.6842, a Δn value of 0.00317, and a R_th_ value of 31.7 nm. In particular, the low-R_th_ feature of the FLPI-1 film might be helpful for the applications in advanced optoelectronic fields.

## Figures and Tables

**Table 1:** Origination and representative research and development of PI materials in the history.

People	Achievement	Affiliation	Ref.
Hermann Staudinger	Discoveries in the field of macromolecular chemistry (Nobel Prize in Chemistry, 1953)	Albert Ludwig University of Freiburg, Germany	[1,2]
Wallace H. Carothers	Establishment of modern polymer science Inventor of polyamide 6,6 (nylon 6,6); Grandfathers of polycondensation chemistry, together with Leo H. Baekeland	DuPont, USA	[3,4]
Paul J. Flory	Moden macrololecular theoretical and experimental physics (Nobel Chemistry Prize, 1974)	DuPont, USA (1934–1938)	[5]
Cyrus E. Sroog	Inventors of practical PIs, together with Walter M. Edwards and Ivan M. Robson et al.	DuPont, USA	[6,7,8]
Jones J. Idris	Earlier report on polycondensation preparation of polypyromellitimides	National Chemical Laboratory, Teddington, England	[9]
John A. Kreuz	R&D of catalysts for chemical imidization of polypyromellitimides (Kapton^®^) film (Lavoisier Medal for technical achievement, DuPont, 1998)	DuPont, USA	[10]

**Table 2:** Inherent viscosities, molecular weights, and solubility of the PI resins.

PI	[*η*]_inh_^a^ (dL/g)	Molecular Weight ^b^			Solubility ^c^				
		*M*_n_ (×10^4^ g/mol)	*M*_w_ (×10^4^ g/mol)	PDI	NMP	DMAc	GBL	CPA	CHCl_3_
FLPI-1	0.73	3.17	5.89	1.85	++	++	++	++	+
FLPI-2	0.64	19.36	32.91	1.70	++	++	++	+	+
FLPI-3	0.99	6.32	12.75	2.02	++	++	++	++	+
PI-ref1	0.77	3.78	6.94	1.83	++	++	++	++	+
PI-ref2	0.67	25.69	41.70	1.62	++	++	++	++	+
PI-ref3	1.02	16.55	29.37	1.77	++	++	++	++	+

^a^ Inherent viscosities measured with a 0.5 g/dL PI solution in NMP at 25 °C; ^b^ *M*_n_: number average molecular mass; *M*_w_: weight average molecular mass; PDI: polydispersity index, PDI = *M*_w_/*M*_n_; ^c^ ++: Soluble; +: partially soluble or swelling; −: insoluble. GBL: γ-butyrolactone; CPA: cyclopentanone.

**Table 3:** Tensile and thermal properties of CPI films.

PIs	Tensile Properties ^a^			Thermal Properties ^b^				
	T_S_ (MPa)	E_b_ (%)	T_M_ (GPa)	T_g, DMA_ (°C)	T_5%_ (°C)	T_max_ (°C)	R_w750_ (%)	CTE (×10^−6^/K)
FLPI-1	112.9	3.5	4.1	436.4	505.5	582.6	67.8	45.8
FLPI-2	150.6	4.5	5.6	422.6	514.4	591.7	65.2	31.8
FLPI-3	158.0	3.1	5.8	422.2	498.7	575.0	65.4	42.8
PI-ref1	113.9	3.8	3.9	401.3	500.7	559.6	65.6	52.0
PI-ref2	149.7	12.6	4.7	376.3	503.1	555.0	53.9	34.4
PI-ref3	175.5	3.8	5.9	381.4	503.1	555.6	55.6	36.1

^a^ T_S_: tensile strength; E_b_: elongation at break; T_M_: tensile modulus; ^b^ T_g, DSC_: glass transition temperatures according to the DSC measurements; T_g, DMA_: glass transition temperatures according to the DMA measurements (peaks of tan δ plots); T_5%_: temperatures at 5% weight loss; T_max_: temperatures at the rapidest thermal decomposition rate; R_w750_: residual weight ratio at 750 °C in nitrogen; CTE: linear coefficient of thermal expansion in the range of 50–250 °C.

**Table 4:** Optical and thermal properties of FDAn-PIs and referenced PI films.

Samples	λ_cut_^a^ (nm)	T_450_ (%)	T_500_ (%)	n_TE_	n_TM_	n_av_	Δn	R_th_ (nm)	L*	a*	b*	Haze (%)
FLPI-1	364	78.6	81.8	1.6852	1.6821	1.6842	0.00317	31.7	94.29	−0.94	5.20	0.73
FLPI-2	355	85.0	86.0	1.6579	1.6188	1.6450	0.03906	390.6	95.04	−0.16	1.20	0.71
FLPI-3	348	85.7	86.1	1.6364	1.6127	1.6285	0.02368	236.8	95.24	−0.07	1.01	0.53
PI-ref1	365	80.0	84.5	1.6395	1.6282	1.6357	0.01131	113.1	94.75	−1.32	5.77	0.64
PI-ref2	346	86.9	87.6	1.6122	1.5525	1.5926	0.05970	597.0	95.52	−0.17	1.11	0.37
PI-ref3	336	87.6	86.8	1.5923	1.5445	1.5766	0.04780	478.0	95.71	−0.02	0.82	0.50

^a^ λ_cut_: Cutoff wavelength; T_450_, T_500_: Transmittance at wavelengths of 450 nm and 500 nm with a thickness of 20 μm, respectively; n_TE_, n_TM_: in-plane and out-of-plane refractive indices of the PI films, respectively; n_av_: average refractive indices of the PI films; Δn: birefringence, Δn = n_TE_ − n_TM_; R_th_: optical retardation, R_th_ = Δn × d, d = 10 μm; L*, a*, b*, see Measurements part.

## Data Availability

Data are contained within this article.
